# Bayesian network approach for reliability analysis of mining trucks

**DOI:** 10.1038/s41598-024-52694-0

**Published:** 2024-02-10

**Authors:** Mohammad Javad Rahimdel

**Affiliations:** Department of Mining Engineering, Faculty of Engineering, University of Birjand, Birjand, Iran

**Keywords:** Mathematics and computing, Engineering

## Abstract

Having a safe and efficient system for mineral transportation is a top priority for all mining operations. Trucks are the most widely used material transportation systems that are applied in both surface and underground mines. Any truck failure disrupts the mineral transportation process and consequently decreases the overall output. Therefore, the reliability analysis of such equipment plays a critical role in increasing the efficiency and productivity of a mining operation. This paper proposes a novel method for analyzing the reliability of a fleet of mining trucks based on the Bayesian Network modeling. Considering the reliability block diagram, the fault tree of trucks was developed according to the logical relationship between the units. Then, a dynamic Bayesian network was constructed according to the conditional probability analysis. Moreover, the relative contributions of each truck’s component to the occurrence of the fleet failure were studied by using critical analysis. The results of this paper show that the successful operation of the fleet of trucks is most sensitive to truck no. 5, which has the highest reliability level in all time intervals. The reliability of the fleet of trucks reaches 0.881 at 20 h, and the fuel injection system of the truck’s engine is the main leading cause of the trucks failure. A proper preventive maintenance strategy should be paid more attention to improve the reliability and availability of the engine system.

## Introduction

The mining sector is one of the most capital-intensive industries. Mining operations have a significant capital cost and operational expenditures. These make mining activities one of the most energy-intensive industries around the world. Therefore, it is crucial to have an efficient, less energy-consuming, labor-intensive, and reliable mining operation. The process of mineral production in hard rock mines is divided into four parts, including drilling, blasting, loading, and hauling. Among them, loading and hauling operations comprise a significant component of the capital and operating costs of mines. Generally, material transportation accounts for about 50–60% of the total mines’ operating costs^1^. This means that mining operation and material transportation are inextricably linked together and it is crucial to use transportation systems with low cost of operation through the entire operational life.

In open pit mines, truck-shovel, belt conveyors, semi-mobile, and mobile crushers are different systems used to transport the broken ore from the working face of a mine to the dumping area. Among these, the truck-and-shovel system is an economical, flexible, and efficient system known as a conventional loading and hauling system^2^. Having more efficient and safe transportation systems leads to operating mines with the lowest operational cost. This requires equipment with less costly downtime and minimal requirement for inspections, servicing, and parts replacement. Unplanned downtimes waste all human and financial resources instead of engaging them to improve production productivity, especially in the most capital-intensive industries such as mining^3^. Heavy-duty dump truck, operated in mines, is a complex vehicle with many interconnecting components or parts. Failure of any subsystem, component, or part will cause a truck to fail. A failed truck disrupts the mineral transportation process and consequently decreases the overall output; therefore, it is crucial to keep the truck at a high level of availability^4^. Understanding the trucks’ complexities, efficiency, and failures helps to fulfill the production goal and reduce unexpected and unrequired costs, increases the machine's lifetime, and results in optimized life cycle costs. This means that all trucks and their subsystems must operate efficiently during their lifetime. In this concept, reliability estimation and prediction of the trucks’ lifetime are necessary to keep them available.

Reliability analysis is a well-known statistical tool to identify the critical components and leading causes of failures in a complex system. In the reliability analysis, high levels of lifetime uncertainty and the risks of failure are estimated, prevented, and controlled^5^. Nowadays, several methods such as fault tree analysis, reliability block diagram, reliability graph, and Markov chain have been developed for equipment fault detection and reliability analysis. Most of the research has been conducted on the failure behavior and reliability of mining equipment and vehicles at different mining operations, including drilling equipment^6^, loaders^7,8^, dragline^9^, and haulage vehicles such as dump trucks^2^ and rolling stocks^10^.

Summit and Halomoan^11^ studied the suitability of the scheduled maintenance program by applying the Weibull distribution for the reliability modeling for Caterpillar 777D dump trucks operating in open pit coal and metal mines in Australia. Results of the mentioned study showed that the suspension system of the truck was the most frequent failing component and the seal failures were the most frequent failure modes. It was mentioned that the front suspension system of the truck had a longer expected life than the rear one. A comparison of the failure data showed that the time to failures at the coal mine site was much less than at the metal mine site for both front and rear suspensions. Regarding the results of the reviewed study, haul road conditions might be the main reason for the low life expectancy of the suspension system that needed to be well maintained. Tumanggor^12^ estimated the reliability of dump trucks in open-pit coal mines in South Kalimantan, Indonesia. Regarding the reliability block diagram approach, the dump truck system decomposed into six subsystems including engine, transmission, hydraulic, electrical, body and frame, and tires. The time between repairs for each system was calculated and then the Weibull distribution function was created to determine the systems’ lifetime. Results of the mentioned study showed that the truck axle had the highest repair time. It was concluded that the broken shaft trunnion had the most reason for the axle damage. He et al.^13^ applied the proportional hazard model (PHM) to predict the reliability of mining trucks. In the mentioned study, the failure of motors’ exhaust valves for 13 trucks was analyzed and then the appropriate reliability model was proposed. Results of the reviewed study showed that the beginning engine hours, barometric pressure, coolant temperature, and fuel temperature had a significant effect on the exhaust valve failures.

In another research, Kishorilal and Mukhopadhyay^14^ studied the reliability of a truck with 85 ton capacity in an open pit mine using the reliability block diagram approach. In this study, the time between failures of a truck engine for three years was used for statistical analysis. The result of the mentioned study showed that the fuel supply subsystem of the motor is more prone to failure than other motor’s subsystems. The time intervals to reach 70%, 80%, and 90% reliability levels were calculated to suggest preventive maintenance intervals for each engine’s subsystems. Angeles and Kumral^15^ proposed a preventive maintenance scheduling for a fleet of dump trucks in an open pit mine in Canada. In the mentioned research, the reliability of each truck was estimated by using the power law process, then optimal inspection intervals were proposed considering the age and the rejuvenation of the trucks after each repair. Wang and Zhang^16^ studied the reliability of truck engines regarding the after-sales maintenance data. In the reviewed research, the main failures were identified, and the lifetime of the diesel engine at different service backgrounds was analyzed and discussed. Results of the studied paper showed that the fuel supply system had the highest number of failures that were inconsistent with the previous studies^14,14,17^. The service background had a considerable effect on the engine’s lifetime. It was also stated that the electronic devices and motor parts working in the high-pressure environment were the leading failure causes of diesel motors. Rahimdel^2^ estimated the residual lifetime of mining truck tires by considering the environmental and operating conditions, including truck axle, working shift, weather conditions, and temperature. The proportional hazard model is used to estimate the remaining useful life of tires for trucks with 100-tonne capacity in an open pit iron ore mine in Iran. Regarding the results of the reviewed study, the temperature had a considerable on the failure behavior of trucks’ tires. In higher temperature levels, the remaining life of tires was in the worst situation. In the mentioned study, checking and inspection intervals were proposed to keep the reliability of tires at a desirable level.

Reviewing the paper, mentioned above, shows that the reliability of truck, operated in the mine, have been studied in numerous previous researches. However, most of these studies evaluated the reliability and failure behavior of a specific part of the truck by using statistical modeling approaches. The application of mathematical models for reliability modeling is a usual approach when historical failure data such as time between failures (TBFs) and time to failures (TTFs) are available. Probability distribution modeling, failure mode and effect analysis (FMEA), and fault tree analysis (FT) are well-known approaches. These methods simplify the reliability analysis processes by using simplified assumptions such as considering only a part of a system, independence of the system components, and availability of failure data. Moreover, these methods couldn’t update the posterior failure probability of the components regarding the evidence data. The main challenge in the reliability analysis of complex systems, with numerous interacting components that might be in an active or failed state, is to represent the interactions between each equipment’s component and their basic and conditional probabilities. This allows us to understand the cause-and-effect relationship between components and accordingly enhance the reliability prediction and evaluation. To address this issues Bayesian Networks (BNs) are found to overcome such challenges^18^. The BNs are used to estimate not only the failure probability of the overall system but also the failure probability of the basic components. They can also be used for robust probabilistic reasoning in uncertain conditions. BNs can also dynamically assess the system performance, considering updated information^19,20^. In recent decades, the BN has been widely used for the reliability analysis of complex mechanical and electrical equipment such as dragline system^21^ complex electronic systems^22^, offshore wind turbines^23^, subsea blowout control systems^24^, wind turbines and diesel generators^25^, and complex railway systems^26^.

Considering that BN are not currently employed for reliability analysis of heavy-duty mining trucks, the contribution of this study lies in implementing a developed reliability modelling approach in a specific case study in Iran. The main aim of this paper is to analyze the reliability and fault diagnosis of a fleet of heavy-duty mining trucks by combining FT and BN. In this approach, it is possible to analyze the root causes of heavy-duty truck failures through fault tree modeling. The FT model provides prior information for BN modelling and the BN is investigate for the reliability analysis and fault diagnosis of a fleet of mineral transportation vehicles with complex components. The system’s components with the high relative contributions to the failure occurrence of the system are identified, as well.

The results of this study are helpful for operation and maintenance managers to find critical components of mining trucks that require further improvement. It is also helpful to propose a suitable maintenance plan to keep the reliability of mining trucks at a desirable level and have a reliable mineral transportation operation. This decreases the operational cost of mineral transportation by maximizing truck availability, as well.

This paper is divided into three sections. Research foundation and the methodology of the paper is described in section “Research methodology”. Application of the dynamic Bayesian network to reliability modelling of the fleet of mining trucks and the critically analysis of the truck’s sub-systems are presented and discussed in section “Application of the BN for the reliability analysis of a fleet of mining trucks”.

## Research methodology

This section is devoted to discussing the theoretical foundation of the paper. The fault tree modeling is presented, and then mapping the fault tree to a Bayesian network is introduced and discussed. Analysis of the components’ role and their importance in the whole system is presented to identify the critical components.

### Fault tree analysis

**Figure 1:**
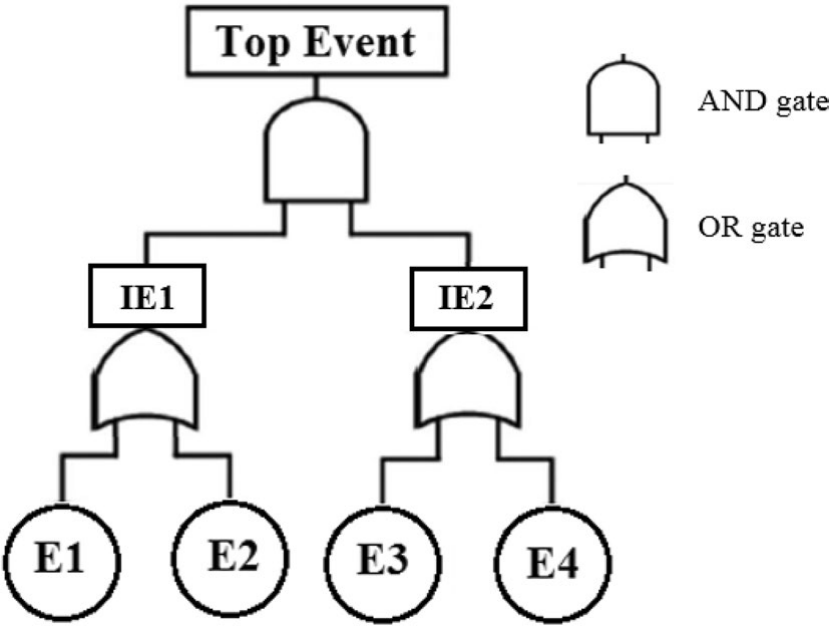
A sample of fault tree.

A fault tree (FT) is a logic diagram in which the potential causes of a failure, called the top event, are postulated. Currently, FT is widely used to study the reliability and risk mitigation of mine hoists^27^, safety and health risks of mining operations^28^, reliability analysis of mining draglines^29^, fault diagnosis of electric trucks^30^, reliability analysis of explosive vehicle^31^, fault diagnosis of road headers^32^, and failure analysis of mine cage conveyance^33^. In this method, the possible ways for the top event to occur are deduced, and the potential causes of this event are evaluated using Boolean logic^34^. The relationships between events in fault trees are represented using gates. Figure 1 shows a sample of the fault tree. AND- and OR- gates are the most widely used ones. The output of OR-gate will occur when any one of all inputs will occur. However, in the case of AND-gate, the output will occur when all inputs occur. For instance, regarding Fig. 1, the intermediate event IE1 will occur when any of the basic events E1 and E2 occur, and the top event will occur when both intermediate events IE1 and IE2 occur.

FT is a top-down method started at a top event and then branches out downwards to display different states of the system by using the logic symbols. After constructing the fault tree, the model is evaluated by quantitative analyses in which the occurrence probability of the primary events is considered for the probability of the top event.

To analyze the reliability by using the approach, mentioned above, basic assumptions of the standard FT analysis are made as follows:

- All basic events in the fault tree model are statistically independent.
- The failures of all components have a constant failure rate. This means that the failures follow the exponential distribution. In this approach, the occurrence probability of basic events at the operating time *t*, is obtained as^35^:
  1 $${P({BE}_{i})}_{t}=1-{\text{exp}}\left(-\lambda t\right)$$

where *λ* is the constant failure rate, and ${P({BE}_{i})}_{t}$ is the occurrence probability of *i*th basic event at time *t*.

- All components are considered as good as new after maintenance operation. On the other hand, the remaining life of a component is independent of its current age.

### Bayesian network modeling

**Figure 2:**
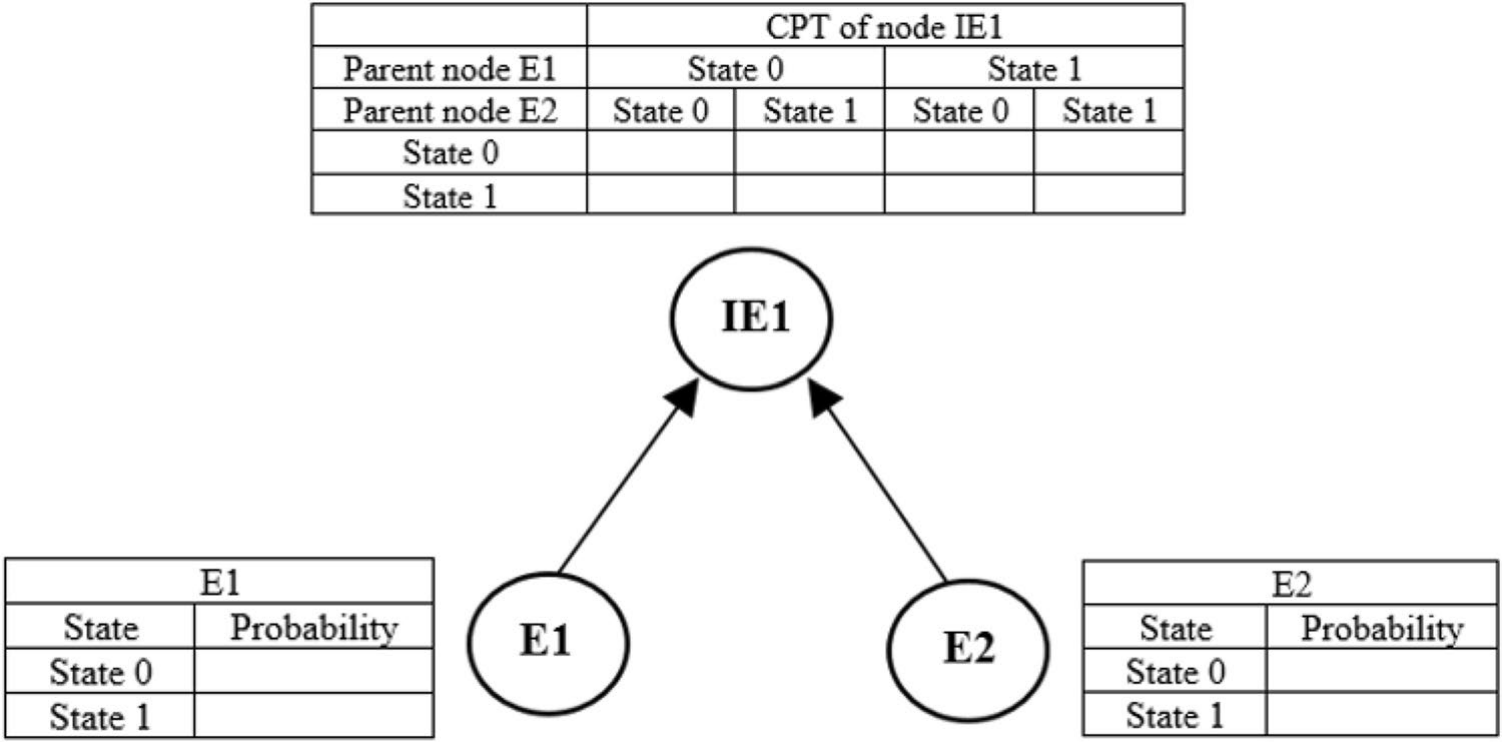
Example of a BN model with two parent nodes and one child node and their corresponding CPT.

Bayesian networks are a sample of graphical models to describe probabilistic relationships within a group of random variables. BNs is a directed acyclic graph, constituted by points (represent variables) and directed edges that connect these points. In a BN, if an arc starts at node A and ends in node B, then node A is the parent of node B. In BNs, a root node is a node without a parent, and a node without any child node is considered a leaf node. Figure 2 shows a simple BN. In this figure, two nodes are connected by a directed edge. The “parent or cause nodes” are the node at the tail (nodes E1 and E2 in Fig. 2), and the “child or effect node” is the node at the head (node IE1 in Fig. 2).

A parent node has only a marginal probability. Each child node in the BN has a Conditional Probability Table (CPT) that illustrates how the states of each node are characterized by the conditional probability for the combination of every state of its parents. In the BN example, shown in Fig. 2, each node has two states: state 0 and state 1. The CPT is used to obtain the joint probability distribution of all the nodes.

Conditional probability is the foundation of the Bayesian reasoning that is expressed as follows^20,23^:

2 $$P\left(A|B\right)=\frac{P\left(A\right)\cdot P\left(B|A\right)}{P(B)}=\frac{P\left(A,B\right)}{P(B)}$$

where $A$ is a hypothetical event or set of hypothetical events, *B* is the observed evidence; $P\left(A|B\right)$ is the posterior probability after observing *B*, $P\left(B|A\right)$ is the conditional probability that *B* is present in every state of A, $P\left(A\right)$ is prior probability before observing *B*, and $P\left(B\right)$ is the marginal probability; and $P\left(A,B\right)$ is the joint probability.

The joint probability distribution function of variables is estimated according to conditional independence and the chain rule in the probability theory. The joint probability distribution of a set of variables ${U=\{X}_{1}\dots . {X}_{n}\}$ can be expressed as follows^20,23^:

3 $$P\left(U\right)=P\left({X}_{1}\dots . {X}_{n}\right)=\prod_{i=1}^{n}P\left({X}_{i}|Pa\left({X}_{i}\right)\right)$$

where $P\left(U\right)$ is the joint probability of a set of variables ${X}_{1}\dots . {X}_{n}$, $P\left({X}_{i}|Pa\left({X}_{i}\right)\right)$ is the conditional probability of ${X}_{i}$, and $Pa\left({X}_{i}\right)$ is the parent nodes of ${X}_{i}$.

The system’s reliability can be obtained through the marginalization of this joint probability distribution. In the reliability analysis via BNs, the prior probability of each event is updated by new information (or posterior probability). When new observation or evidence $E$ is given, the posterior probability can be calculated by conditional probability function or Bayesian inference as follows^23,36^:

4 $$P\left(U|E\right)=\frac{P(U,E)}{P(E)}=\frac{P\left(E|U\right)\cdot P(U)}{P(E)}=\frac{P\left(E|U\right)\cdot P(U)}{\sum_{E}P\left(E|U\right)\cdot P(U)}$$

### Mapping the fault tree to the Bayesian network

**Figure 3:**
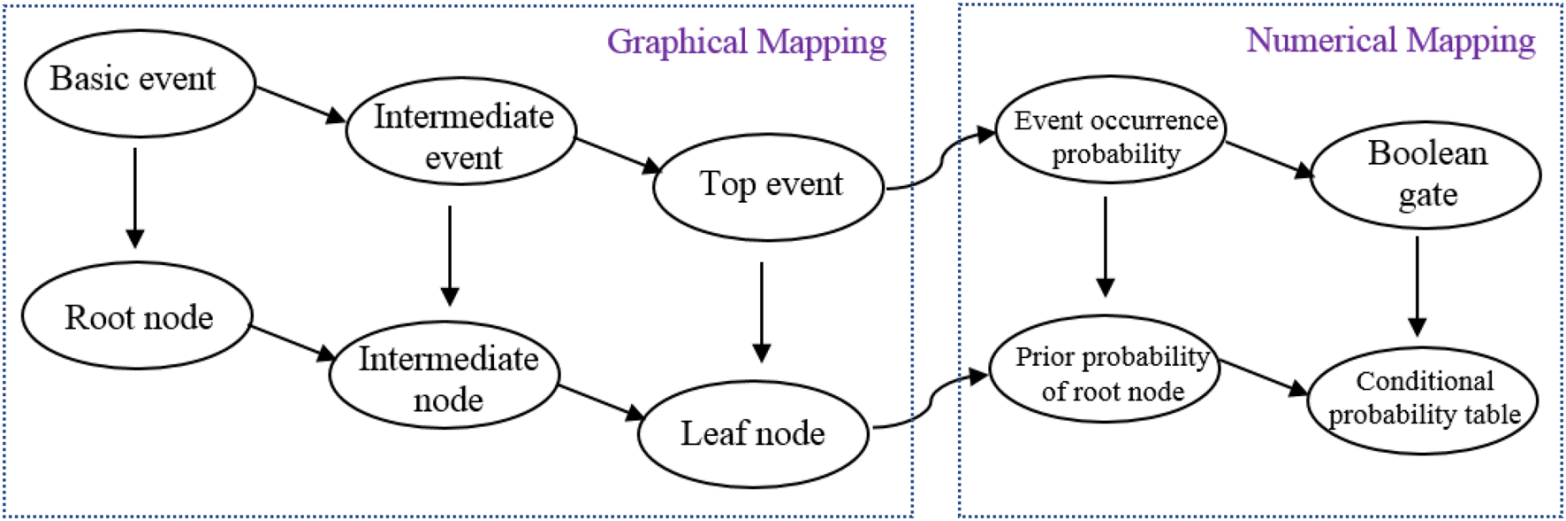
Process of mapping the fault tree to the Bayesian network.

According to the constructed fault tree model of the system, the corresponding Bayesian network can be obtained by mapping the fault tree to the Bayesian network. In graphical mapping, basic and intermediate events in the FT are considered the root and intermediate nodes in the corresponding BN, respectively. Moreover, the top event in the FT is the leaf node in the BN. The occurrence probabilities of the primary events are assigned to the corresponding root nodes, and the conditional probability table is developed for each child node^37,38^. The process of Mapping FT to BN is shown in Fig. 3.

### Dynamic reliability analysis

After mapping the fault tree to the Bayesian network, the predictive analysis is done on the model to obtain system reliability. To achieve this, the estimated occurrence probability of basic events at the specific operating time is used as the quantitative input in BN modeling to conduct dynamic probability reasoning. In this approach, the reliability of the system can be obtained as follows^23^:

5 $$R\left(t\right)=1-{{\text{Pr}}\left\{TE\right\}}_{t}$$

where $R\left(t\right)$ is the system reliability at time *t* and ${{\text{Pr}}\left\{TE\right\}}_{t}$ is the top event occurrence probability at time *t*.

### Criticality analysis

Identification of the most critical components of a system that require more attention leads to reducing the frequency of failures and accordingly improves the performance of the whole system. The critical analysis is used to identify the role of components and their importance in the occurrence of the top event. On the other hand, criticality analysis is used to show the systems’ reaction when the input parameters of a component are different from the others. There are different importance measure methods to measure the relative importance of basic events in FT that can be applied to the basic nodes of BN, in a parallel way. In this paper, the Birnbaum Importance Measure (*BIM*) and Risk Reduction Worth (*RRW*), as the most common importance measures, are used to carry out the sensitivity analysis^37,39^.

The *BIM* in a BN can be obtained as follows^40^:

6 $${I}_{{RN}_{i}}^{BIM}=P\left(LN|{RN}_{i}=1\right)-P\left(LN|{RN}_{i}=0\right)$$

where ${I}_{{RN}_{i}}^{BIM}$ is the Birnbaum importance measure of root node ${RN}_{i}$, $P\left(LN|{RN}_{i}=1\right)$ is the conditional probability of the leaf node for the occurrence of root node ${RN}_{i}$, and $P\left(LN|{RN}_{i}=0\right)$ is the conditional probability of the leaf node for nonoccurrence of root node ${RN}_{i}$.

The *RRW* indicates the effect of root node on the leaf node concerning non-occurrence of root node. In a BN the *RRW* can be calculated as follows^41^:

7 $${I}_{{RN}_{i}}^{RRW}=\frac{P(LN)-P\left(LN|{RN}_{i}=0\right)}{P(LN)}$$

where ${I}_{{RN}_{i}}^{RRW}$ is the *RRW* importance of root node ${RN}_{i}$, $P(LN)$ is the occurrence probability of leaf node, and $P\left(LN|{RN}_{i}=0\right)$ is the conditional probability of the leaf node for nonoccurrence of root node ${RN}_{i}$.

## Application of the BN for the reliability analysis of a fleet of mining trucks

Trucks are an essential part of the mine haulage system that must be available for a given mission time. This section aims to apply the proposed methodology, mentioned in section “Research methodology”, for analyzing the reliability of a fleet of mining trucks in Golgohar Iron Mine, Iran. Golgohar Iron deposit is located in 55 km southwest of Sirjan City, Kerman Province, Iran. Golgohar iron ore complex is located in six different anomalies encompassing a 10 × 4 km area. Among them, anomaly no. 3 with a mineral reservoir of more than 660 million tons is the largest iron ore reserve. Regarding the exploration studies, the total ore reserve of this anomaly is calculated as 616 million tons with an average grade of 54.3% Fe. Golgohar no. 3 mine is the biggest iron ore mine in Iran, and its ore extraction is more than 15 million tons per year. After the blasting operation, broken rocks are transported to crushers by Komatsu dump trucks with 65-tonne capacity and Caterpillar dump trucks with 100-tonne capacity. Currently, 28 dump trucks with 65-tonne capacity and 20 trucks with 100-tonne capacity are working three shifts per day^2^.

The remaining part of this section is devoted to the reliability analysis of a fleet of mining trucks through the Bayesian Network analysis. A fleet of six Caterpillar 777 trucks with 100-tonne capacity, TR1 to TR6, were selected for data gathering and analysis. The failure data were gathered over one year. To study the reliability of trucks, it is crucial to identify the components and subsystems that have the most significant effect on the reliability. Therefore, at the first step, the dump truck is decomposed to its sub-systems/component as given in Table 1.

**Table 1:** ID, systems, and sub-systems of the dump truck.

System/sub-system		Component		ID
Engine system (ES)	Engine major (ES1)	Crankcase		ES 1.1
		Cylinder head		ES 1.2
		Engine block		ES 1.3
		Turbocharger		ES 1.4
		Oil pump		ES 1.5
		Air manifold		ES 1.6
	Fuel injection (ES2)	Pipes and connectors		ES 2.1
		Exhaust		ES 2.2
		Fuel Pumps		ES 2.3
		Injectors		ES 2.4
		Sensors		ES 2.5
		Other		ES 2.6
	Engine cooling (E3)	Pipes and connectors		ES 3.1
		Water pump		ES 3.2
		Cooling fan		ES 3.3
		Radiator		ES 3.4
	Engine electrical system (ES4)	Switches and sensors		ES 4.1
		Starter		ES 4.2
		Battery		ES 4.3
		Alternator (Dynamo)		ES 4.4
Drive system (DS)	Brake (DS1)	Brake hydraulic system (DS 1.1)	Retarder	DS 1.1.1
			Hoses and connectors	DS 1.1.2
			Hydraulic valves	DS 1.1.3
		Brake pneumatic system (DS 1.2)	Relief valve	DS 1.2.1
			Air compressor	DS 1.2.2
			Air hoses and connectors	DS 1.2.3
			Other	DS 1.2.4
		Brake block		DS 1.3
	Steering (DS 2)	Oil pump		DS 2.1
		Switch and sensors		DS 2.2
		Pipe and connection		DS 2.3
		Shock absorber		DS 2.4
Transmission system (TS)		Gear Box		TS 1
		Rear drive shaft		TS 2
		Front drive shaft		TS 3
		Power take-off unit		TS 4
		Planetary gear		TS 5
		Connectors		TS 6
Tire (Tr.)		Front tire		Tr. 1
		Rear tire		Tr. 2
		Wheels and rims		Tr. 3
Body and chassis (BC)	Cab (BC 1)	Air condition (BC 1.1)	Cooler compressor	BC 1.1.1
			Fan	BC 1.1.2
			Air lines and connections	BC 1.1.3
			Water pipe and connections	BC 1.1.4
		Seat		BC 1.2
	Body hoist (BC 2)	Hydraulic hoist pump		BC 2.1
		Pipes and connections		BC 2.2
		Hoist cylinders		BC 2.3
	Chassis			BC 3
Electrical system (El.)		Cables		El. 1
		Switches and sensors		El. 2
		Lights		El. 3

**Figure 4:**
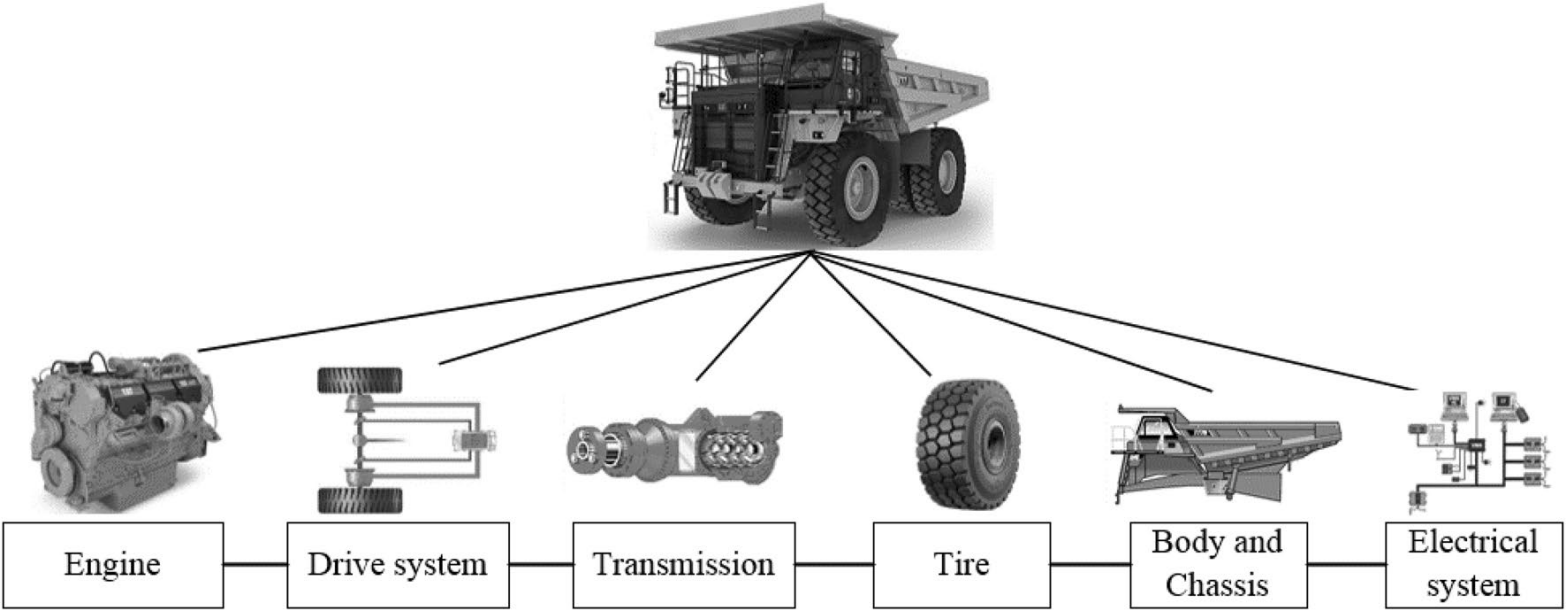
Reliability block diagram of the studied truck.

Construction of the reliability block diagram (RBD) is necessary for evaluating the reliability of trucks. The RBD is a graphical representation of a system components from a reliability viewpoint. The reliability block diagram of the studied truck is shown in Fig. 4. Regarding Fig. 4, the truck is considered to be a system that consists of seven major sub-systems including engine, drive system, power transmission, tire, body and chassis, and electrical sub-systems.

**Figure 5:**
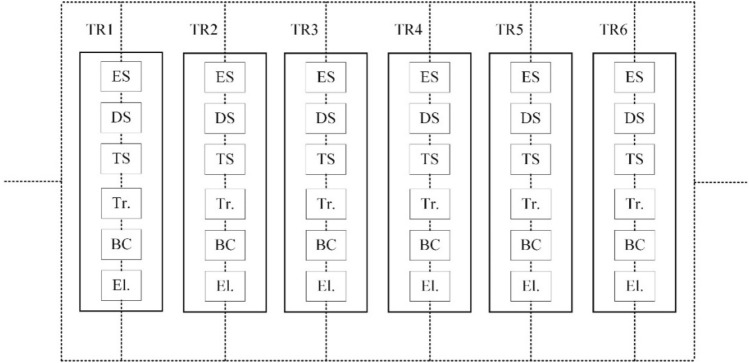
Block diagram of the fleet of mining trucks.

**Figure 6:**
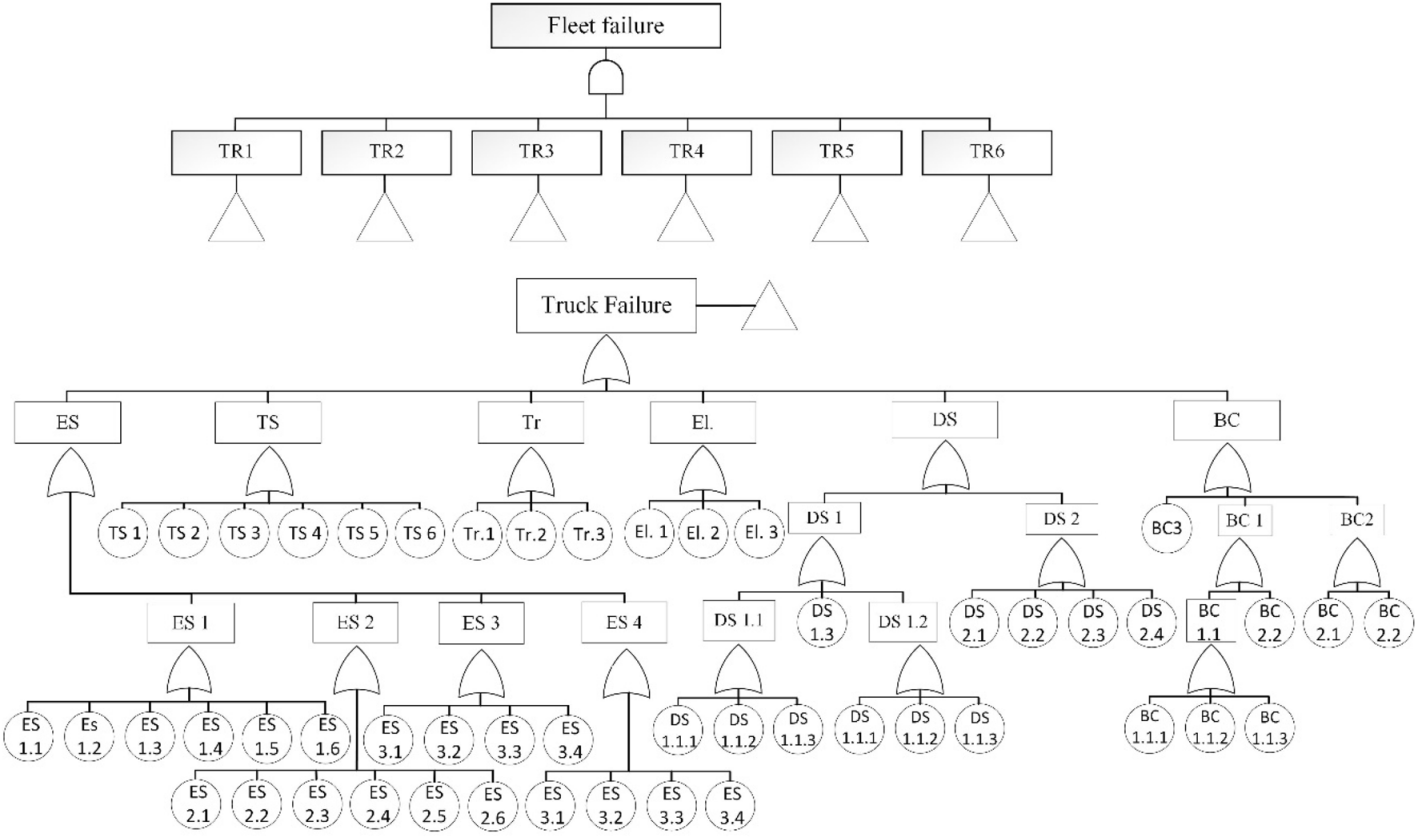
Fault tree of fleet of trucks.

Regarding the block diagram of all in-operation trucks of the mine as a serial-parallel network (Fig. 5), the Bayesian network analysis is performed to complete the reliability analysis. To identify all possible causes of failure and to indicate critical sub-systems concerning the failure of interest, a fault tree was developed according to RBD and shown in Fig. 6. To perform a reliability analysis, it is necessary to access the historical failure data to obtain the failure rate of the basic event. Regarding the basic assumption of a standard fault tree, the failures of all components have a constant failure rate. It should be noted that, the constant failure rate of each basic event is calculated as the reverse of mean time between failures (MTBF)^42^. Thus, the probability of basic failures with an identified failure rate can be obtained. The required failure data were gathered over 9 months from the operation and maintenance unit of the mine. For example, the time between failure data of the engine’s component of truck TR1 were obtained and given in Table 2. Accordingly, the failure rate of each basic event of truck TR1 has been computed and given in Table 3.

**Table 2:** Sample of TBF data of the engine’s component of the truck TR1.

No. of failure	TBF (h)	No. of failure	TBF	No. of failure	TBF	No. of failure	TBF
1	12	6	43	11	756	16	58
2	22	7	587	12	1294	17	478
3	99	8	755	13	399	18	74
4	227	9	366	14	1257	19	323
5	207	10	435	15	1486	20	895

**Table 3:** Failure rate of the basic events for truck TR1.

ID	Failure rate	ID	Failure rate	ID	Failure rate	ID	Failure rate
ES 1.1	0.0008	DS 1.2.1	0.0021	ES 2.1	0.0084	ES 4.2	0.0025
ES 1.2	0.0031	DS 1.2.2	0.0022	ES 2.2	0.0030	El. 1	0.0056
ES 1.4	0.0026	DS 1.2.3	0.0050	BC 1.2	0.0015	El. 3	0.0030
ES 1.5	0.0010	DS 2	0.0024	ES 2.3	0.0058	El. 4.1	0.0030
BC 2.1	0.0059	DS 2.4	0.0107	ES 2.4	0.0019	ES 4.2	0.0025
BC 2.2	0.0050	ES 4.1	0.0030	ES 3.1	0.0028	ES 4.3	0.0005
Tr. 1	0.0012	BC 1.1.1	0.0053	BC 1.1.4	0.0065	El. 4.4	0.0010
Tr. 2	0.0039	BC 1.1.2	0.0010	TS 1	0.0018	TS 4	0.0013
Tr. 3	0.0014	BC 1.1.3	0.0016	TS 2	0.017	TS 5	0.0036

### Reliability analysis of the fleet of trucks

**Figure 7:**
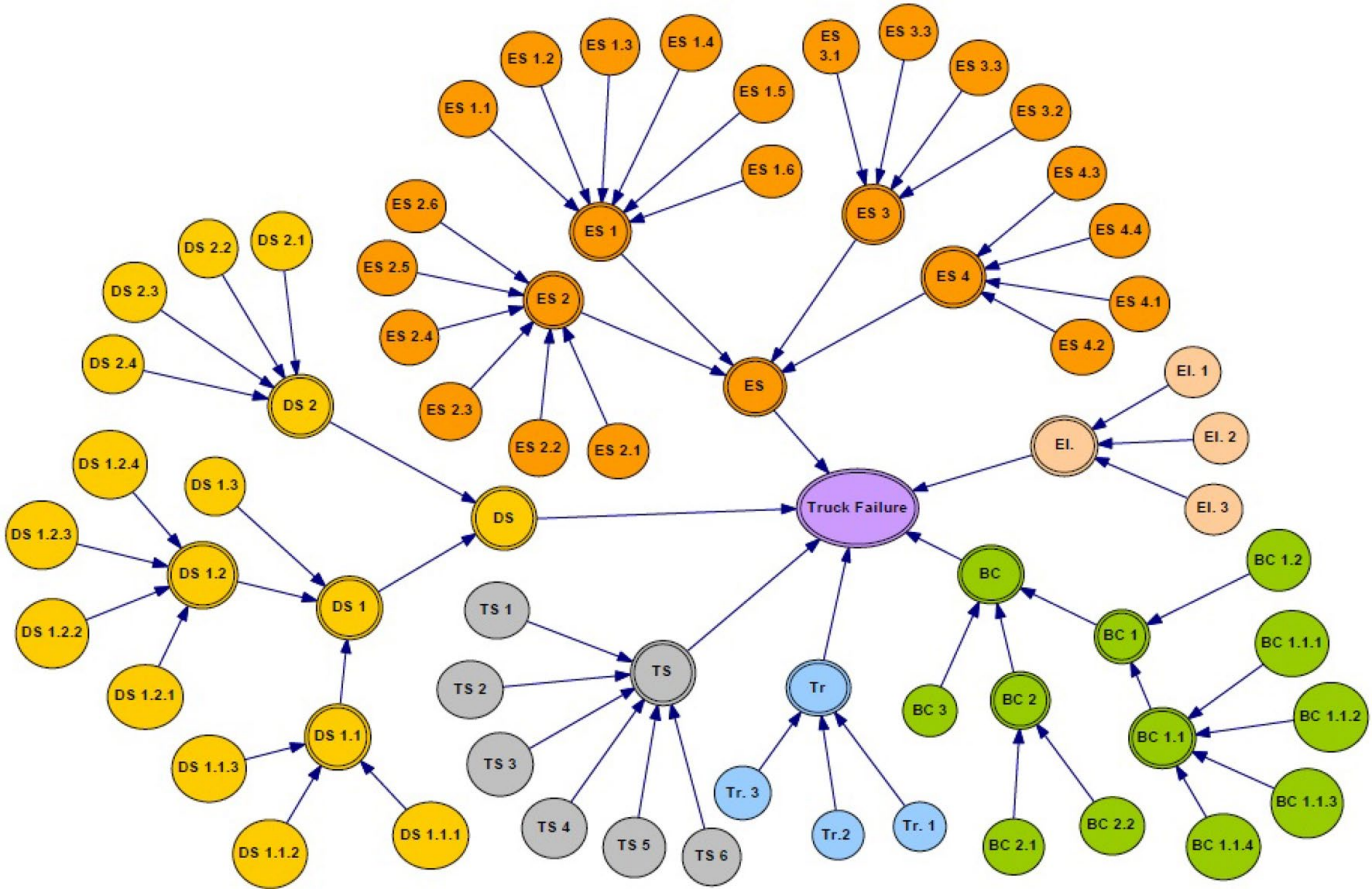
Bayesian network for the studied truck.

To perform the reliability analysis using BN, first, the fault tree is transformed to BN. As mentioned in section “Mapping the fault tree to the Bayesian network”, the mapping process has two parts, including graphical mapping and numerical mapping. In the graphical mapping part, the basic events connected with the intermediate node of the FT can be mapped with corresponding intermediate nodes in the BN. Then, the top event in the fault tree can be converted into leaf nodes in the Bayesian network. In the quantitative mapping process, the occurrence probabilities of the basic events are mapped to the priory probabilities of the BN. In this way, the probability of the gates can be mapped into the conditional probability tables in the Bayesian network. The BN for the studied trucks is presented in Fig. 7. In Fig. 7, the nodes with different systems have been shown with different colors. Regarding Fig. 6, there are 17 parent nodes. They are DS, DS 1, DS 2, DS 1.1, DS 1.2, ES, ES 1, ES 2, ES 3, ES 4, BC, BC 1, BC 1.1, BC 2, Tr, Ts, and El. The remaining are child nodes with their own CPT associated with their parent nodes. The failure probability of each root node was obtained from the corresponding failure rate, and then, the CPT was assigned to nodes. In this paper, GeNIe software^43^ was used to model the Bayesian network and determine the most critical basic events affecting truck failure.

**Figure 8:**
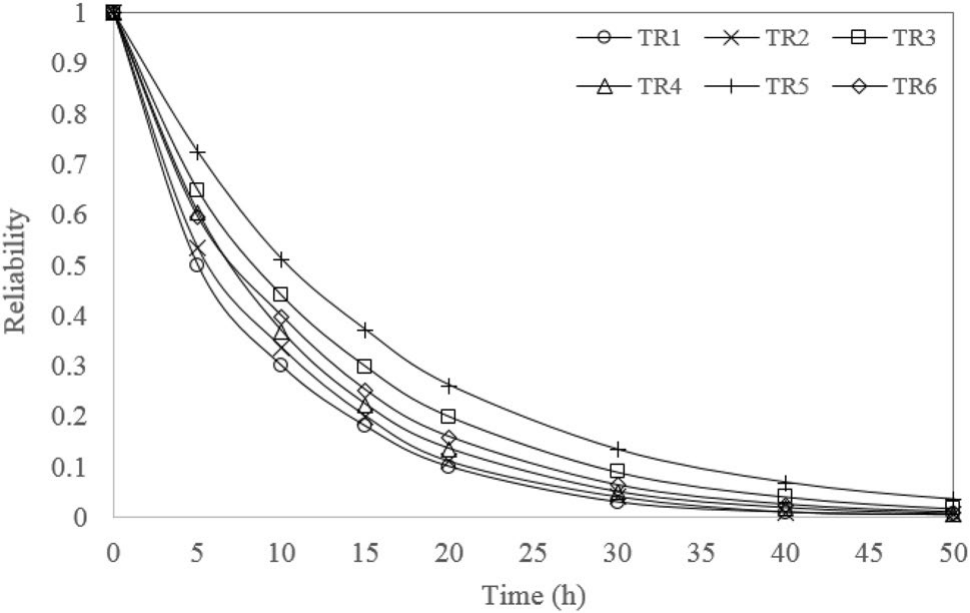
Reliability of all studied trucks.

**Figure 9:**
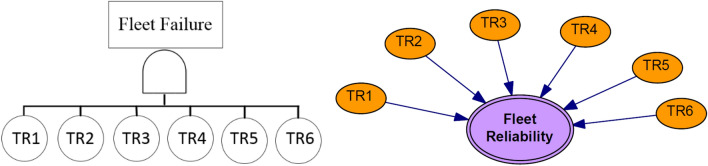
Fault tree and corresponding Bayesian network for a fleet of six trucks.

**Figure 10:**
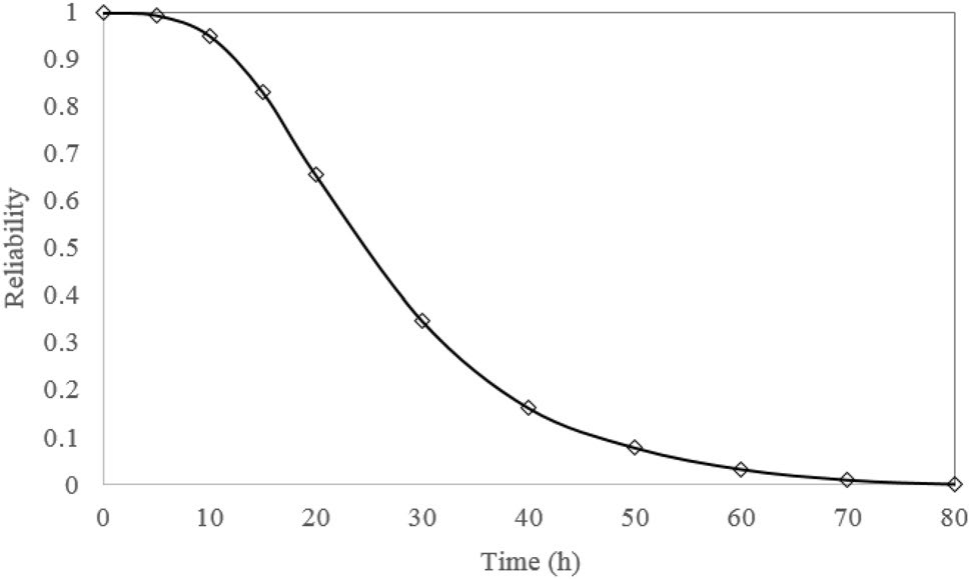
Reliability of the fleet of trucks.

According to the failure rate of the basic events, the failure probabilities were obtained in different time intervals. Then, the GeNle program is employed to use failure probabilities for modeling the Bayes Network. The network was updated by using the failure probability of basic events at different observation times to compute the failure probability of the truck and accordingly its reliability. In this approach, the reliability of each truck at different observation times was obtained using the predictive inference of the corresponding BN. The results are given in Fig. 8. A similar process, mentioned in section “Mapping the fault tree to the Bayesian network”, was applied to obtain the reliability of a fleet of trucks. As at least one of the trucks must operate for the mineral haulage system to succeed, a fleet of trucks can be considered a parallel system. The FT and corresponding BN of a parallel system with six trucks is constructed and shown in Fig. 9. The reliability of the fleet of trucks at different time was obtained using the BN and shown in Fig. 10.

### Critically analysis of the truck sub-systems

**Figure 11:**
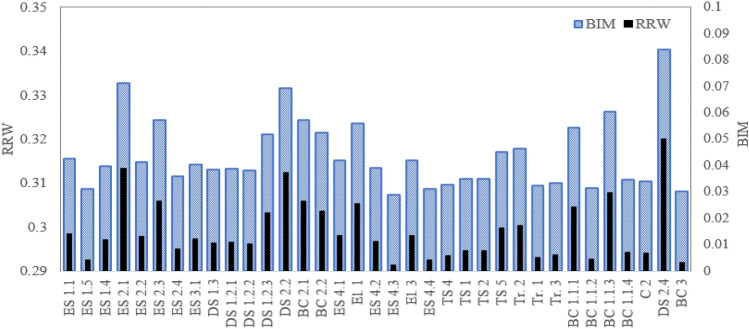
Importance sore of components for truck TR1.

**Figure 12:**
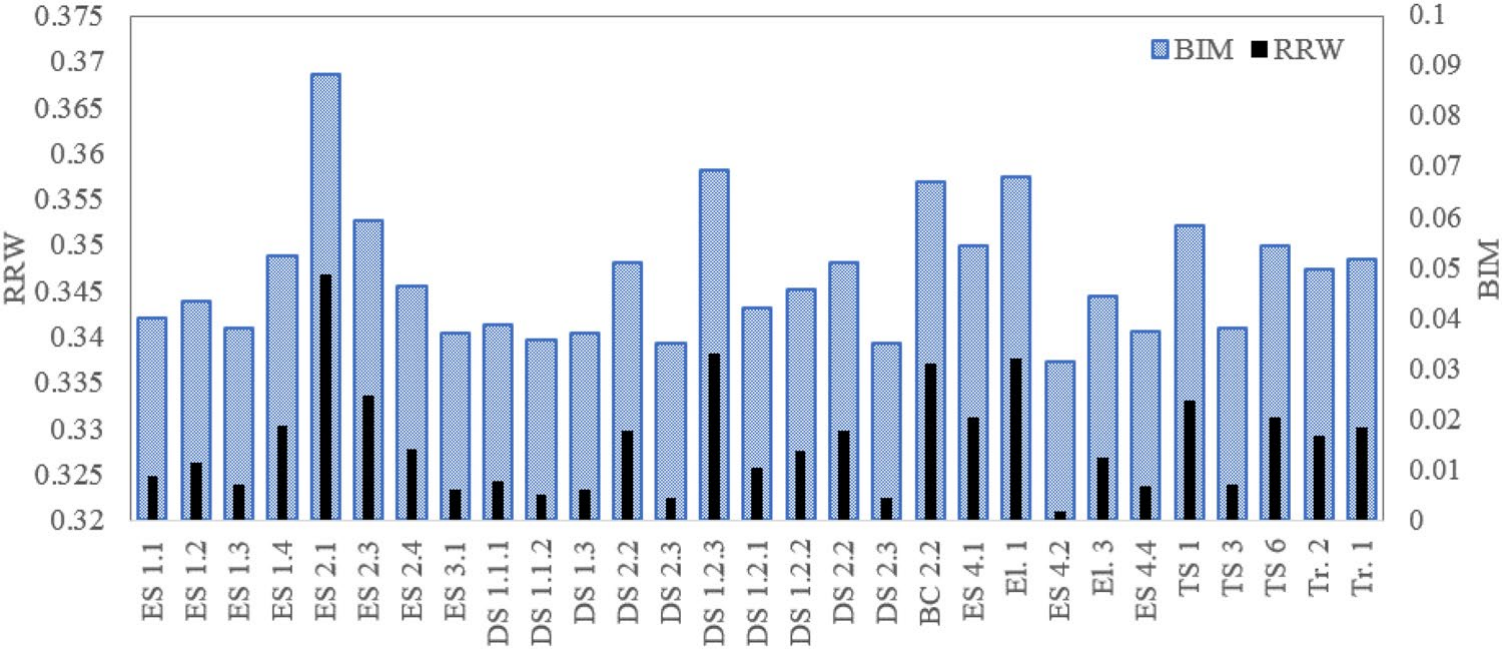
Importance sore of components for truck TR2.

**Figure 13:**
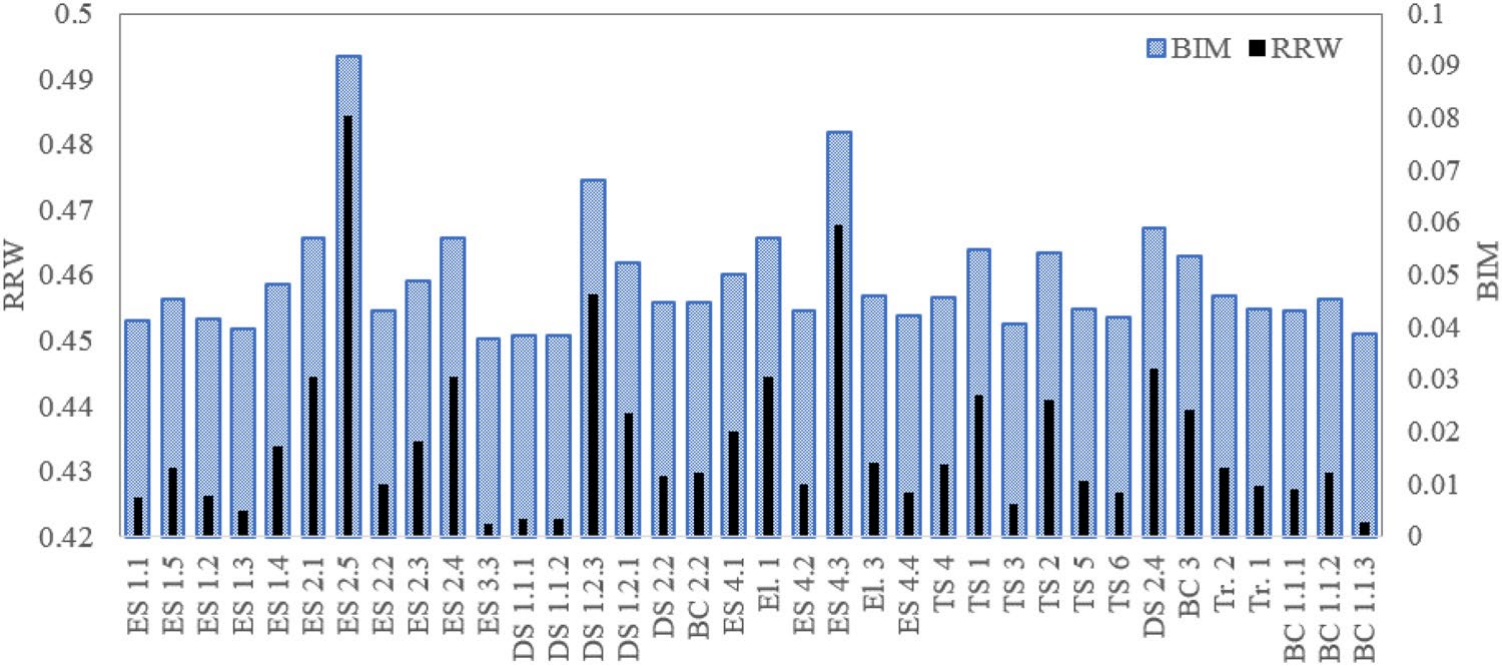
Importance sore of components for truck TR3.

**Figure 14:**
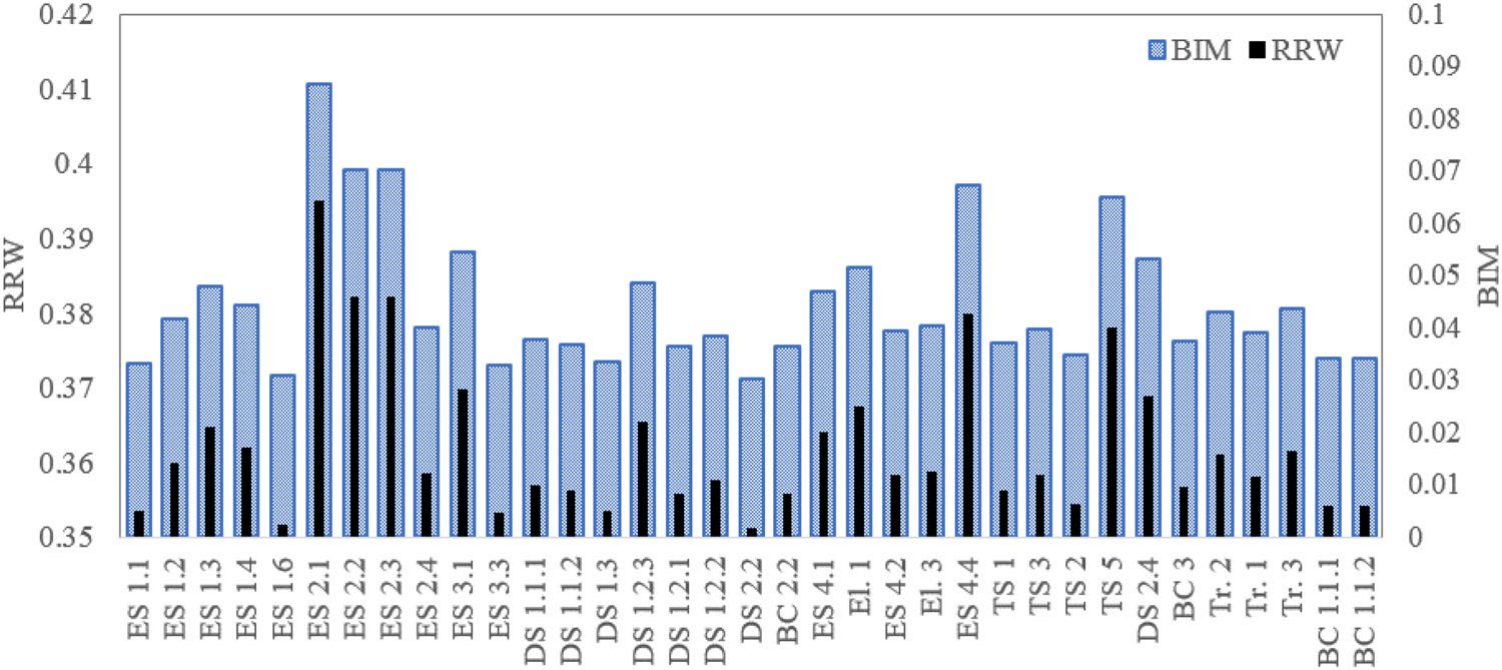
Importance sore of components for truck TR4.

**Figure 15:**
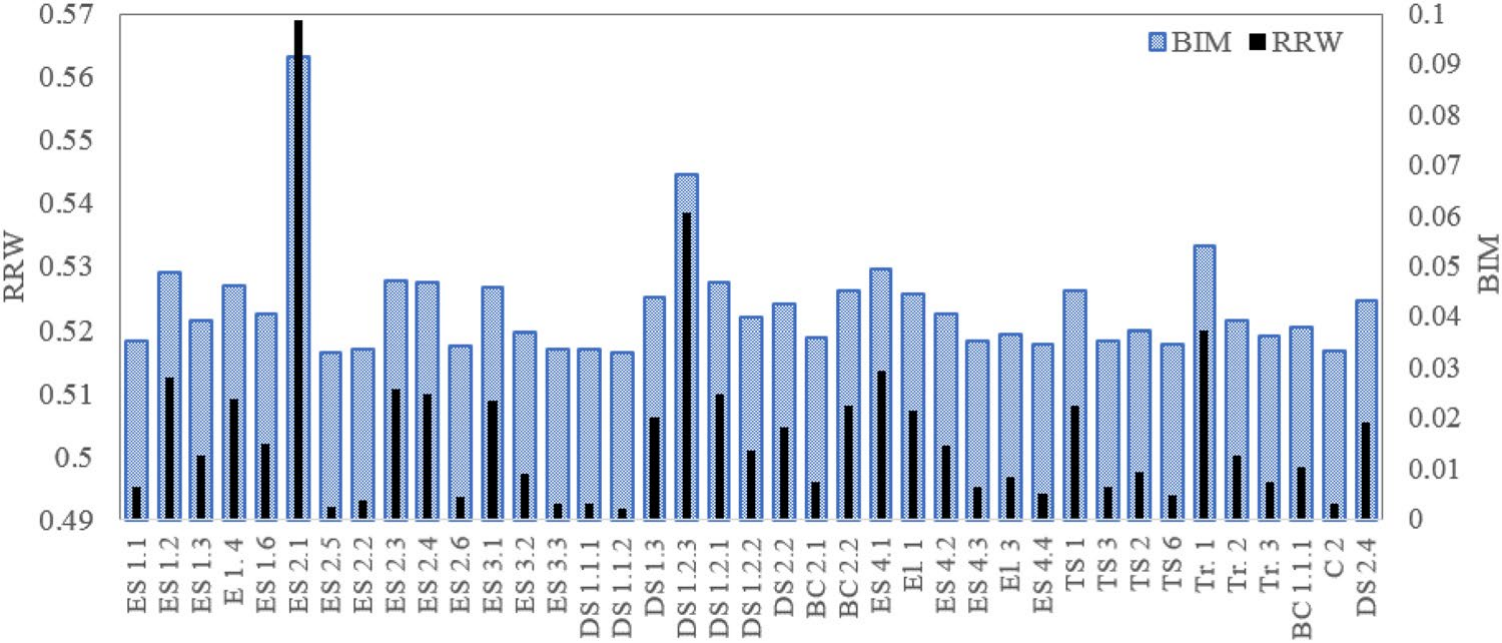
Importance sore of components for truck TR5.

**Figure 16:**
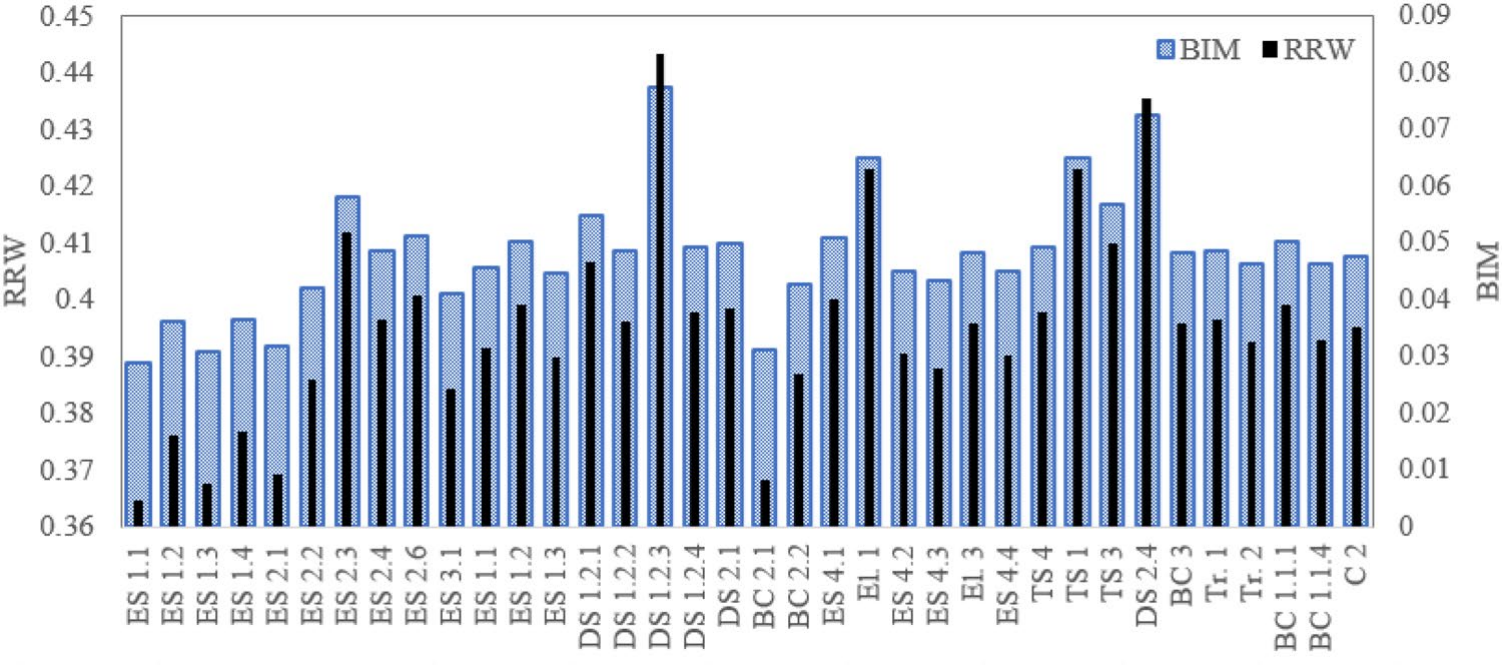
Importance sore of components for truck TR6.

One of the main aims of the reliability analyses is to identify those components/systems/subsystems that are critical from the reliability perspective. This helps decision-makers to take appropriate actions to prevent failures and mitigate consequences. To achieve this, in this paper, the relative importance of each basic node of the BN is performed by using the Birnbaum importance measure (BIM) and risk reduction worth (RRW) as described in Eqs. (5) and (6). Figures 11, 12, 13, 14, 15, and 16 shows the values of BIM and RRW importance measures for trucks TR1 to TR6. Moreover, the relative contributions of each truck to the occurrence of the failure probability of the material haulage system are performed. The results are given in Table 4.

**Table 4:** Importance sore for each truck in the haulage fleet.

Truck	*I* (BIM)	*I* (RRW)	Rank
TR1	0.0072	0.0003	6
TR2	0.0147	0.0079	5
TR3	0.0195	0.0127	2
TR4	0.0174	0.0106	3
TR5	0.0248	0.0180	1
TR6	0.0170	0.0102	4

## Discussion

Regarding the reliability analysis of the studied trucks, although truck no. 5 has the highest reliability compared the others, the reliability of this truck degrades from 1 to 0.7241 only after 5 h. The reliability of trucks TR1 and TR2 maintains a lower value over 50 h. As a result, special attention should be paid to these two trucks during the daily inspection and maintenance. The reliability of all trucks reaches zero approximately in 50 h. Preventive maintenance should be implemented on all trucks if high reliability is expected. Reliability analysis of the fleet of trucks indicates that the reliability of the fleet of trucks maintains a high value over 10 h. The fleet reliability degrades to 0.952 during 10 h period. This means that after these times onwards, the failure probability of the haulage fleet increases to 4.8%. The reliability of the haulage fleet reaches zero in 80 h.

Regarding the critical analysis, the shock absorber, pipes, and connectors of fuel injection and switch and sensors of the steering system are the most critical root nodes for the failure occurrence of the truck TR1. Regarding the results, pipes and connectors of the fuel injection systems have the most contribution to the failure occurrence of trucks TR2 that is consistent with past studies^14,16,17^. In the following order, air hoses and connectors of the brake pneumatic system, pipes and connections of the hydraulic body hoist system, and gearbox have a high relative importance on the occurrence of the failures. As seen from Fig. 13, sensors of the fuel injection system, battery, and the air hoses and connectors of the brake pneumatic system are recognized as the most critical reasons for the failure of truck TR3. Figures 14 and 15 show that pipes and connectors of the fuel injection systems are the most critical components of the trucks TR4 and TR5. These results are consistent with previous studies^14,14,17^. From Fig. 16, air hoses and connectors of the brake pneumatic system, shock absorber, and gearbox are the most critical components of truck TR6, respectively.

The relative contributions of each truck to the occurrence of the fleet failure probability show that truck TR5 is the most critical truck in the mineral transportation fleet. These results are inconsistent with the results of the reliability analysis. The reliability of the fleet of trucks is most sensitive to truck TR5 because this truck has the highest reliability compared to other trucks.

Reliability modeling is an appropriate approach for preventive maintenance (PM) planning to minimize operation-stopping breakdowns. In this approach, PM intervals are estimated considering the reliability level we wish to have in our operation. In many engineering operations, 80% reliability assures both efficiency and performance^6,10^. In this study, considering 80% as the target reliability, the reliability-based PM intervals for all trucks were obtained and given in Table 5. Regarding Table 5, all subsystems of trucks should be subjected to PM activities at their planned time interval. However, to optimize the maintenance program, all tasks with similar intervals can be carried out in one acceptable interval to all related subsystems. The combined PM intervals for all trucks’ subsystems were calculated using this approach and given in Table 6. As can be seen from Table 6, the drive system of truck TR1 and the body and **c**hassis of trucks TR1 and TR2 have the shortest PM interval and should be inspected every 7 h. The transmission system of trucks TR5, and TR6 and tire of truck TR6 have the highest maintenance intervals and should be serviced and inspected in 33 and 60 h, respectively. After 11 h operation, the engines of trucks TR1, TR2, and TR4, the drive system of trucks TR5 and TR6, and the electrical system of all trucks except TR5 should be checked and serviced. The engine system of trucks TR3, TR5, and TR6, the drive system of trucks TR2, TR3, and TR4, and the transmission system of trucks TR1, TR2, TR3, and TR4, and all trucks tires except truck TR 6 should be checked and inspected every 16 h. After 24-h operation, the body and chassis of trucks TR3, TR4, TR5, and TR6 and the electrical system of trucks should be serviced and maintained together.

**Table 5:** Reliability-based PM interval for 80% reliability level.

Subsystem	Truck					
	TR1	TR2	TR3	TR4	TR5	TR6
Engine system	12.35	11.53	14.56	9.84	20.22	14.56
Drive system	6.18	15.17	15.83	18.20	13.48	11.74
Transmission system	16.48	11.03	20.2	13	33.09	33.06
Electrical system	11.16	8.47	14	11.74	24.27	9.58
Body and Chassis	10.18	3.43	24.27	28	33.09	26
Tires	15.73	10.71	26	15.17	19.16	60.66

**Table 6:** The improved PM intervals for each truck’s subsystem.

Subsystem	Truck	Combined PM interval (h)
Engine system	TR1, TR2, TR4	11
	TR3, TR5, TR6	16
Drive system	TR1	7
	TR2, TR3, TR4	16
	TR5, TR6	11
Transmission system	TR1, TR2, TR3, TR4	16
	TR5, TR6	33
Electrical system	TR1, TR2, TR3, TR4, TR6	11
	TR5	24
Body and chassis	TR1, TR2	7
	TR3, TR4, TR5, TR6	24
Tires	TR1, TR2, TR3, TR4, TR5	16
	TR6	60

Such information is helpful in identifying the weakest part of the truck that is most likely to cause material transportation failure directly. These are also helpful for maintenance and operation management to improve the availability level of the trucks.

## Conclusions

Application of Bayesian networks in the field of reliability analysis has increased during the last decade because this is an efficient method for analysis of the complex systems. In this paper, the dynamic Bayesian network approach is used for the reliability analysis of a fleet of mining trucks as a case study: Golgohar Iron Mine, Iran. To achieve this, the truck description was directly mapped to a Bayesian network by using GeNIe tools. In this approach, the truck failure probability was automatically calculated, and all relevant elements that affect the performance of the fleet of the mineral transportation system were identified. The results of this paper can be summarized as follows;

- Reliability of trucks TR1 and TR2 maintains a lower value in comparison to other trucks and the special inspections and maintenance activations should be considered for these trucks.
- Reliability of the fleet of trucks degrades to 0.972 after 10 h and the failure probability of the fleet of trucks reaches one at about 80 h.
- The fuel injection system of the truck’s engine is the most critical system that has the most contribution to the failure occurrence of the mineral transportation fleet.
- Truck TR5 is the most critical truck in the mineral transportation fleet of the Golgohar iron ore mine and requires less service and maintenance than other trucks.
- According to the reliability-based maintenance optimization program, the proposed maintenance interval for the critical subsystems such as the engine, drive, power transmission, electrical, body and chassis, and tire are 13.5, 11, 24.5, 17.5, 15.5, and 38 h on average, respectively.

The results if this study help the maintenance planners and managers in the determination of better maintenance tasks and the prevention of catastrophic failures. It is also helpful to find the weak points of the system that significantly affect the reliability behavior of the system. However, considering the effect of the environmental factors and operational conditions such as haul road quality, weather condition, and operator skill level that influence the failure behavior of trucks’ subsystems and components was the limitation of the current study and therefore, a separate BN can be created to study the reliability of trucks under the operational conditions. Another limitation of this study is that the created BN could not model the statistical dependencies between the basic events which may lead to inaccurate results.

## Data Availability

The data presented in this study are available on request from the corresponding author.
